# Creating sustainable health care systems: Agreeing social (societal) priorities through public participation

**DOI:** 10.1108/JHOM-02-2018-0065

**Published:** 2019-03-18

**Authors:** Peter Littlejohns, Katharina Kieslich, Albert Weale, Emma Tumilty, Georgina Richardson, Tim Stokes, Robin Gauld, Paul Scuffham

**Affiliations:** 1 Department of Primary Care and Public Health Sciences, King’s College London, London, UK; 2 Department of Political Science, University of Vienna, Vienna, Austria; 3 School of Public Policy, University College London, London, UK; 4 Department of General Practice and Rural Health, University of Otago, Dunedin, New Zealand; 5 School of Business, University of Otago, Dunedin, New Zealand; 6 Centre for Applied Health Economics, Griffith University, Southport, Australia

**Keywords:** New Zealand, Evidence-based practice, Hospital management, Inequality, Health services sector, National Health Service

## Abstract

**Purpose:**

In order to create sustainable health systems, many countries are introducing ways to prioritise health services underpinned by a process of health technology assessment. While this approach requires technical judgements of clinical effectiveness and cost effectiveness, these are embedded in a wider set of social (societal) value judgements, including fairness, responsiveness to need, non-discrimination and obligations of accountability and transparency. Implementing controversial decisions faces legal, political and public challenge. To help generate acceptance for the need for health prioritisation and the resulting decisions, the purpose of this paper is to develop a novel way of encouraging key stakeholders, especially patients and the public, to become involved in the prioritisation process.

**Design/methodology/approach:**

Through a multidisciplinary collaboration involving a series of international workshops, ethical and political theory (including accountability for reasonableness) have been applied to develop a practical way forward through the creation of a values framework. The authors have tested this framework in England and in New Zealand using a mixed-methods approach.

**Findings:**

A social values framework that consists of content and process values has been developed and converted into an online decision-making audit tool.

**Research limitations/implications:**

The authors have developed an easy to use method to help stakeholders (including the public) to understand the need for prioritisation of health services and to encourage their involvement. It provides a pragmatic way of harmonising different perspectives aimed at maximising health experience.

**Practical implications:**

All health care systems are facing increasing demands within finite resources. Although many countries are introducing ways to prioritise health services, the decisions often face legal, political, commercial and ethical challenge. The research will help health systems to respond to these challenges.

**Social implications:**

This study helps in increasing public involvement in complex health challenges.

**Originality/value:**

No other groups have used this combination of approaches to address this issue.

All health care systems are facing ever greater demands due to aging populations and increasing opportunities to intervene within finite resources. In order to create effective, fair and sustainable health systems many countries are introducing ways to prioritise health services which involves making difficult decisions concerning who gets (and who does not get) health care interventions. Priority setting requires technical judgements of clinical effectiveness (what works) and cost effectiveness (is it worth the money). But these judgements are embedded in a wider set of social (societal) value judgements that underlie justifiable reasoning about priorities, including fairness, responsiveness to need and non-discrimination, and obligations of accountability and transparency. Even when these decisions are based on the best available evidence generated by technically sound health technology assessment (HTA) programmes they frequently face legal, political, methodological, philosophical, commercial and ethical challenges. One author’s experiences as the founding Clinical and Public Health Director of the National Institute for Health and Care Excellence (NICE) 1999–2012 in the UK (responsible for priority setting within a HTA framework) has reinforced his view that there is a need to undertake more research in how to develop approaches to include social values into the prioritisation process that were conceptually clear and, most importantly, easy to apply on a routine basis in a consistent manner. This is necessary to reassure patients and the public that institutions making tough prioritisations decisions on their behalf were doing so in an acceptable robust manner and reflected societal values. Stimulating stakeholder involvement, especially public participation, is considered key to making difficult decisions acceptable to the public and professionals as well as fulfilling the moral obligation to make the process as democratic as possible. It will only happen if the public see the relevance of their contribution, how their input is to be achieved and the output assessed.

This paper describes a multidisciplinary research programme over the last seven years that has addressed the twin-related challenges of how to reflect societal values in health prioritisation and how to encourage the public to understand the need for health prioritisation and to get involved in these difficult decisions.

## A new approach based on the application of social (societal) values

Over the last seven years, an international collaboration of ethicists, philosophers, political scientists, public health practitioners, lawyers, health economists and health technologists has worked together to address this challenge and develop solutions to the problems. This has been carried out through regular multidisciplinary group meetings in London and a series of international workshops applying mixed methods. In 2011, the inaugural workshop was convened at Gresham College London to initiate the process by exploring how social values were currently being incorporated into decision-making processes in different countries. This event was supported by the Wellcome Trust and the Nuffield Trust. Representatives from Germany, France, South Korea, the USA, China, World Bank and South America attended the event. The debate was informed by a working document on social values prepared by Professor Albert Weale (Weale and Clark, 2012) and each delegate described how social values were applied to prioritisation processes in their country according to the framework. The underlying ethical issues relevant to a framework approach were fully explored and enhanced the view that both content and process values were important (Biron *et al.*, 2012).

## A social values framework

A social values framework emerged from the deliberations of the workshop that consisted of both content and process values (Littlejohns, Sharma and Jeong, 2012; Littlejohns, Yeung, Weale and Clark, 2012; Littlejohns, Weale, Chalkidou, Teerwattananon and Faden, 2012). The process aspects build upon Daniels’ and Sabin’s theory of accountability for reasonableness (A4R) (Daniels and Sabin, 2008). A4R emphasises the importance of process values such as transparency and accountability given that reasonable people might disagree on the content and the outcome of difficult prioritisation decisions. The new framework, however, emphasises the importance of addressing content as well as process values, reflecting a broader ethical view of HTA (Bombard *et al.*, 2011; Abelson *et al.*, 2007) and aligning itself with a “rights” perspective. It suggests that content values such as clinical and cost effectiveness, fairness and quality of care are equally important in bringing about fair prioritisation decisions as process values. Transparency about the process of making a prioritisation decision is meaningless if it does not provide an indication of the arguments, criteria and trade-offs that ultimately led to the final outcome. Transparency may, in some cases, even have the opposite of the intended effect if documents or process descriptions that are made available in the public domain are too long, or too complex, to be evaluated by a non-expert audience. Process values provide the lens through which to evaluate the content of the decision-making process.

## Testing the framework and creating a decision-making audit tool (DMAT)

This framework was first tested on three national institutions (Littlejohns, Sharma and Jeong, 2012; Littlejohns, Yeung, Weale and Clark, 2012; Littlejohns, Weale, Chalkidou, Teerwattananon and Faden, 2012) which demonstrated that it was possible to interrogate institutions’ value profiles through publicly available data. The first workshop had demonstrated that the social values framework could be usefully applied in different countries: the UK (Littlejohns, Sharma and Jeong, 2012; Littlejohns, Yeung, Weale and Clark, 2012; Littlejohns, Weale, Chalkidou, Teerwattananon and Faden, 2012), Germany (Kieslich, 2012), the USA (Keren and Littlejohns, 2012), China (Docherty *et al.*, 2012) South Korea (Ahn *et al.*, 2012) Australia (Whitty and Littlejohns, 2014), Thailand (Tantivess *et al.*, 2012) and South America (Cubillos *et al.*, 2012). As part of a National Institute for Health Research funded research project funded through its Collaboration for Leadership in Applied Health Research and Care South London programme the framework was converted into a DMAT (Kieslich and Littlejohns, 2015) in order to facilitate its use in assessing institutions and collecting data. For each value, a series of questions was developed to support the review of an institution. The user would then grade how complete that value was reflected in the institution’s documentation and policies (Table I).

**Figure 1:**
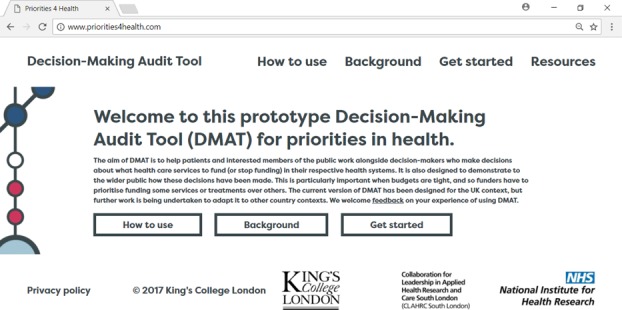
Picture of DMAT website

In order to be useful, any decision tool needs to be valid (actually measures what it purports to) and reliable (consistent over time and between observers). Different types of validity tests are applied, for example, face validity ascertains that the measure appears to be assessing the intended construct under review. Stakeholders are well placed to assess face validity, since they can assess the extent to which the criteria are relevant to decision making. Although this is not the classic “gold standard” type of validity, it is an essential component in enlisting motivation of stakeholders. Against this background, a multi-stakeholder workshop of 40 UK health care managers, health professionals, commissioners, patients and the public was convened in London to introduce the decision tool and to apply it to local health prioritisation decisions. In light of their comments, the tool was revised, replacing the domain of solidarity with quality of care for use in a UK setting. Workshop participants felt strongly that the value of “solidarity” was not as pertinent in the English NHS context as what was incorporated by it was already included in the value of “fairness”. Instead, the participants proposed that “quality of care” needed to be included in a framework that assesses CCG decision making, not just because of recent national scandals in poor quality health provision, but also because it reflects what the public, patients and their carers care about. To assess criterion-related validity (the extent to which a measure is related to an outcome) the framework is currently being applied to a national sample of CCGs and we will be triangulating the results with interviews of local stakeholders and publicly available measures of CCG performance published by the Department of Health (Kieslich and Littlejohns, 2015). Following this preliminary piloting, it was decided to convert the DMAT into an interactive online version. This would facilitate more widespread use of the DMAT and, because data would be automatically captured (through a digital registration process), provide a mechanism to eventually assess inter observer reliability. Working with a design company an interactive digital online version has been developed which consists of a series of questions that will allow internal and external audit of how an institution is incorporating values into its decision making (www.priorities4health.com/) (Figure 1).

Finally, the DMAT was piloted in a non-UK setting – New Zealand. The DMAT has been applied to a selection of key national and district health agencies to ascertain whether the information required is available and why, while also exploring whether the DMAT tool is relevant to the New Zealand context and what adjustments may be required. Furthermore, this research facilitates consultation with Health Agency Stakeholders regarding: whether an explicit evaluation of a process’ fairness will affect public perception of the decision, regardless of outcome, for any one individual; and how well public voice/social values are explicitly included in decision making at present. Consultation with representatives of the public voice have begun with more extensive work planned, to ascertain: whether they agree an explicit evaluation of a procedural fairness will affect their perception of a decision regardless of outcome; how their voices or values could be included in decision making where it currently is not; and whether they find the DMAT tool criteria sufficient/relevant/applicable. Stakeholders who saw transparency and engagement with the public as largely positive reasoned that this was due to the opportunities to gain knowledge, relationship building and internal improvement. Those stakeholders that perceived it as negative, however, explained that inclusion could lead to bad press (e.g. a group disadvantaged by a decision), misunderstandings and futility in improving perceptions. This is despite many organisations being required to have consumer panels, have public meetings, share materials and the like. The DMAT with some modifications could be an appropriate tool in the New Zealand setting, along with more work to address responsiveness to Māori (the indigenous population who have treaty-based specified rights), effective models of engagement and a prescription for organisations around transparency.

## Public participation as the key to acceptable prioritisation

It is generally considered that public participation in prioritising health decisions is an essential prerequisite for acceptable prioritisation decisions. The Organisation for Economic Cooperation and Development (Caddy and Vergez, 2001) – in recognising the importance of engaging citizens in policy making – considers the likely benefits of greater participation to include increasing the chances of successful implementation of a policy: reinforcing the legitimacy of the decision-making process and its final results; increasing the chance of voluntary compliance; and increasing the scope for partnerships with citizens. But evidence demonstrating how exactly it should be done and how effective it is on a routine basis is lacking (Church *et al.*, 2002; Ocloo and Mathews, 2016; Bruni *et al.*, 2008; Daniels *et al.*, 2018). Moreover, in practice, what the public involvement contributes is often under-theorised and may be different for different instances or stages of decision making. What power does the public hold in their role within the decision-making exercise? Are they adding further perspectives in order to better inform decision making without making a decision *per se*, or are they adding further “votes” in the decision making itself, so that any given decision reflects an aggregate of varied views?

Despite these concerns public participation in decision making is being encouraged and has become a key policy in many countries. For example, in the UK, Health Authorities are legally required to now routinely seek the views of local people and communities in the assessment of health services and interventions when setting health care priorities. Under the New Zealand Public Health and Disability Act 2000, national- and district-level agencies are legally required to have community and public health advisory committees. In Australia, the National Health and Hospitals Reform Commission has recommended that a systematic mechanism be developed to formulate health care priorities that incorporate community perspectives as well as economic and clinical considerations. However, we still have a long way to go translate these policy aspirations into routine activity in a manner that is efficient and acceptable to the public. While there are a few evaluations of specific approaches in the UK and New Zealand, e.g. elected board members (Gauld and Horsburgh, 2014) more methodical and targeted research on a range of current approaches combined with the development of new and innovative approaches using novel technology will be the best way forward.

To help address this deficit, we convened a second multidisciplinary workshop in 2016 with delegates from 16 countries and international organisations to identify how the public are currently engaged with the prioritisation processes in different countries and to explore how more effective participation could be encouraged. A special edition of five papers was published on the conference deliberations. In the introduction, Weale *et al.* (2016) described how a mixed-methods approach based on a literature review and a conceptual discussion revealed the common themes emerging in the field of public participation and health priority setting. They concentrated on public participation that is collective in character, which is relevant to whole groups of people and not single individuals. They concluded that the rationales for public participation can be found in democratic theory, especially as they relate to the social and political values of legitimacy. They propose in light of the empirical evidence presented during the workshop that public participatory activities such as protests and demonstrations should no longer be labelled “unconventional” but should instead be labelled as “contestatory participation”. This is to better reflect a situation in which these modes of participation have become more widespread and acceptable in many parts of the world. In a further conceptual paper, Weale (2016) went on to explore this concept in more depth and noted that discussions of public participation and priority setting typically presuppose certain political theories of democracy, and he explored two theories: the consensual and the agonistic. He took a theoretical reconstruction approach of two ways of thinking about public participation in relation to priority setting in health care, drawing on the work of Habermas (1996), a deliberative theorist, and Mouffe, a theorist of agonism (Laclau and Mouffe, 1985). He concluded that different theoretical approaches can be associated with different ways of understanding priority setting. These two theories are not the only theoretical alternatives there is often a merged middle ground. For example, among deliberative theorists there are competing views about the extent to which mini publics need to aim at consensus, with some, like Gutmann and Thompson (1996, 2004), urging that “deliberative theory can be formulated in cases of persistent and deep moral disagreement”. Similarly, among some agonistic theorists, like Tully (2005, 2008), open deliberation is a way of dealing with difference. However, the sharply contrasting character of deliberative and agonist democracy in the work of Habermas (1996) and Mouffe (2005) provides a perspective that makes it possible to see more clearly how different modes of public participation may be best theorised. Habermas noted that we cannot expect democracy in health policy to compensate for the lack of democracy in the political system at large. The third paper (Slutsky *et al.*, 2016) summarised data from 12 countries, chosen to exhibit wide variation, on the role and place of public participation in the setting of priorities. It exhibited cross-national patterns in respect of public participation, linking those differences to institutional features of the countries concerned. The approach is an example of case-orientated qualitative assessment of participation. The paper drew on a unique collection of country case studies in participatory practice in prioritisation, supplementing existing published sources. In showing that contestatory participation plays an important role in a sub-set of these countries it has broadened the debate about public participation in priority setting beyond the use of mini publics and the observation of public representatives on decision-making bodies’ practices. It concluded that no system has resolved the conceptual ambiguities that are implicit in the idea of public participation. The fourth paper (Kieslich, Bump, Norheim, Tantivess and Littlejohns, 2016; Kieslich, Ahn, Badano, Chalkidou, Cubillos, Hauegen, Henshall, Krubiner, Littlejohns, Lu, Pearson, Rid, Whitty and Wilson, 2016) offered a different perspective concentrating on one common clinical therapeutic challenge and assessed how public participation had been approached in four countries and its effect. New hepatitis C medicines such as Sofosbuvir highlight the complex and dynamic interaction between the striving for innovation, disputed clinical evidence, budget impact and fairness in health priority setting. The paper examined the role of public participation in addressing these considerations. It employed a comparative case study approach in Brazil, England, South Korea and the USA assessing the differences and similarities in the coverage decisions about the antiviral Sofosbuvir and how the public and patients were involved in the decision-making processes. The main public participation issues were the role of the universal right to health in Brazil, the balance between innovation and overall budget impact in England, the effect of dubious medical practices on public perception in South Korea and the role and the acceptability and legitimacy of priority setting processes in the USA. The analysis suggested that public participation contributes to raising attention to issues that need to be addressed by policymakers. Public participation activities can thus contribute to setting policy agendas, even if that is not their explicit purpose. However, whether policymakers see this as a legitimate function of such groups remains to be seen. The final paper (Hunter *et al.*, 2016) concluded that challenges emerge as a result of problems of both consensus and contestatory modes of public involvement in health priority setting. At least two recurring themes emerged. The first was the importance, but also the challenge, of establishing legitimacy in health priority setting. The differing country experiences suggest that we understand very little about the conditions under which differing types of participation generates sufficient legitimacy to be influential. A second observation was that public participation takes a variety of forms that depend on the structures in a given national context. Given this variety the conceptualisation of public participation needs to be expanded to take account of local culture. The paper concluded that the challenges of public involvement are closely linked to the question of how legitimate processes and decisions can be generated in priority setting. This suggests that future research must focus more narrowly on conditions under which legitimacy is generated in order to expand the understanding of public involvement.

## Developing novel ways of stimulating public participation and understanding

One of the main challenges of prioritisation processes which goes to the heart of its legitimacy is when treatment is withheld from an individual or a group that is considered “needy”. This type of “case study” is nearly always newsworthy and the system is easily portrayed as “being unfair” and often triggers an adverse popular response. This is also being reflected in the examples from Stakeholders in the New Zealand project (Tumilty *et al.*, 2018). What is missing is the counter argument that highlights that should the “needy” patient be allocated treatment, then due to the opportunity cost, many more patients would miss out on treatment and suffer. However, it is difficult to make a “cause celebre” out of these unknown patients. One potential approach is to take those cases that make the public consciousness but use them to explore issues in a more subtle thorough way than is usually adopted by the popular press. This is a way of drawing the public’s attention to the need to prioritise health services fairly in a manner that makes sense and is relevant to stakeholders, patients and the public. In the UK, we are exploring the medium of film to explore new ways of interacting with the public. One author has collaborated with young film makers from the KCL Entrepreneurship Institute to produce a health prioritisation film relevant to the UK. The film is “The lottery of Devolved Cancer Care” (www.youtube.com/watch?v=DVhvzr97-ZM) (this is the 20-min version) and there is 40-min version with more patient interviews (www.youtube.com/watch?v=7dz0gXw5i64). The film uses variation in access to expensive cancer drugs in the four home countries of the UK (health care is a devolved responsibility for England, Wales, Scotland and Northern Ireland). It is based on the circumstances that led a cancer patient Irfon Williams moving from Wales to England to get his treatment. He established a charity to raise the issues of differential access to treatment. Irfon died three months after being interviewed and before he could see the final film but Becky, his widow, said on viewing it, “I think it is beautifully filmed and thought provoking to those who are outside this bubble of cancer treatment”. His autobiography was published in May 2018 (www.amazon.co.uk/Fighting-Chance-Autobiography-Irfon-Williams/dp/1845276779). In the film, he highlights very specifically that he accepts that not all treatment can be available but considers that a fair process needs to be in place. If this was the case then he feels that patients, even if they did not receive their treatment, would accept that priority setting is inevitable. This is an aspect that was not covered at all during the many hours of news coverage of his circumstances and will be used to raise awareness of the need for public participation in these processes. The film is to be presented in a series of workshops in England together with the DMAT to explore the prioritisation issues that are most important to the public. The resulting deliberation will be assessed by an ethnographer and feed into a new programme to encourage public participation in local and national health prioritisation processes. We intend to extend this approach to create a range of country-specific films addressing issues of fairness in health care prioritisation. Two are already planned for New Zealand and Australia and we are exploring future films in Thailand and Chile.

## Prioritisation as a way to achieve universal health coverage (UHC): the essential contribution of public participation

While all health care systems are facing rising challenges in creating sustainable health systems this is particularly so for those countries seeking to implement UHC. Following endorsement by the World Health Organization (WHA, 2005; WHO, 2010), The World Bank (2015) and the United Nation’s Sustainable Development Goals (WHO, 2014), the drive towards UHC is now one of the most prominent global health policies. Addressing the challenges inherent in such a massive undertaking is involving politicians, academics, practitioners, patients and members of the public from a myriad of conceptual perspectives. Despite the multiplicity of approaches, two have emerged to dominate current discourse. The first is a “rights-based” approach and the second is referred to as “priority setting”. Protagonists of the first, such as Boaz (Boaz *et al.*, 2014), suggest that this should be the dominant paradigm. The right to health is enshrined in the United Nations (UN) Declaration of Human Rights (UN, 1948) and further established in the UN International Covenant on Social Economic and Cultural Rights (UN, 1966), which defines “the right to the highest attainable standard of health”. This legally binding Covenant also sets out States Parties obligations to protect, respect and fulfil the right to health. General Comment (UN, 2002) on the right to health, while not legally binding, provides robust guidance on the implementation of the right to health. Boaz argued that a “rights-based” approach to health (inclusive of health care and the social determinants of health and underpinned by core principles including accountability, participation and non-discrimination) embraces the social, political and economic context in which people experience health and all other approaches should fit into this movement.

The “priorities” approach is underpinned by “HTA” which is defined by the WHO as the systematic evaluation of properties, effects and/or impacts of health technology. It is a multidisciplinary process to evaluate the social, economic, organisational and ethical issues of a health intervention or health technology. The main purpose of conducting an assessment is to inform a policy decision making. Supporters of this approach argue that, although at its heart the “rights-based” approach is a noble aspiration, its implementation can actually lead to a diminution in health (Easterly 2009) when courts are involved (Mœstad *et al.*, 2011; Norheim and Wilson, 2014) to enforce human rights law. When countries progress towards UHC, they are forced to make difficult choices about how to prioritise health issues and expenditure: which services to expand first, whom to include first and, in effect, who gets treatment and who does not. A further WHO report (WHA, 2005; Chan, 2016) from a rights perspective provides guidance about how countries can address these issues through three principles that should inform choices on the path to UHC: first, coverage should be on the basis of need, with extra weight given to the underprivileged; second, the aim should be to generate the greatest improvement in health; third, contributions should be based on ability to pay, and not need. However, the report does not address how a country should fulfil its moral and legal obligations regarding the right to health with their obligations to set health priorities fairly. This question is pertinent even for countries who have largely achieved UHC. While those most passionate advocates on both sides consider priority setting and the right to health will always lead to contradictory policies many now think that they can be made to work together to promote individual and public health (Rumbold *et al.*, 2017). In a Lancet editorial, the authors highlight three key factors that would ensure synergy. First, those responsible for advising on or ensuring a fair allocation of health care resources (e.g. priority setters and local and national health planners), and those charged with upholding the right to health (e.g. legislators and judges), need to recognise broader and more recent interpretations of each approach (Hunt, 2016). Priority setting is not only about a utilitarian drive to maximise health benefits across the population, nor is the right to health about securing every individual’s access to health care regardless of cost. Second, when substantive and procedural principles for ensuring fair allocation of resources devoted to health have been decided through a transparent and participatory process, states should institutionalise priority setting. This could include an institution for assessment of new and health technologies, an advisory committee for wider questions of allocative efficiency and fairness, and action on the social, economic, and political determinants of health. Such bodies must be accountable to their populations, the government and the judiciary. Ensuring the proper functioning of these institutions should be recognised as one way in which states contribute to the implementation of the right to health. Third, when an acceptable interpretation of the content of the right to health under national law has been clarified, finance ministers should review their budgets, considering the state’s obligations under that right. The right to health, just as civil and political rights, requires resources, to function whether through taxation or other means. As with civil and political rights, failure to uphold these rights should result in judicial review.

## Practical approaches to getting the public’s view

Time will tell if this guidance gains traction in the international health policy world. However, whatever their differences, both approaches agree that public participation is key feature of both governance structures. Yet, while there has been a tremendous drive to stimulate public participation in making these tough decisions over the last 20 years, it is apparent that the policy aspiration of public involvement has raced ahead of the establishment of an evidence base underpinning it (Abelson *et al.*, 2013). Researchers are now seeking to address this issue (Scuffham *et al.*, 2014) but it seems that the policy-level debate between a Humanities/Legal and Methodological/Health Economic approach at a national policy level described in the previous section is also being re-enacted at a research level in the field of public participation (Boaz *et al.*, 2014; Burton *et al.*, 2014). In a commentary on an extensive research programme comparing citizen councils and discrete choice methods to elicit public priorities and preferences in two key areas for health care – the use of emergency services and management of obesity (Harris *et al.*, 2015, 2018; Scuffham *et al.*, 2016, 2018; Whitty *et al.*, 2014) – Boaz refers to the argument that researchers often focus on the hardware of participation (the how to, methods, approaches, guidelines, etc.) rather than the “software” of values, norms and codes that shape scientific practice (Wilsdon *et al.*, 2005) and goes onto suggest that all these initiatives should be viewed from a “rights” perspective. Burton *et al.* (2014) respond by highlighting that their research is comparing two frequently used methods of public participation and offers further evidence of the potential for deliberative events, such as citizen’s juries, to provide excellent opportunities for “ordinary citizens” to engage in complex health policy debates. However, they also observe that this is an expensive process and it is difficult to imagine it being applied on a very wide scale to the full panoply of contemporary health policy concerns. They suggest that one way forward is to move away from the notion that the active involvement of all citizens in all decisions that might affect them is the pinnacle of participation in practice, and a more modest set of questions should be asked every time a participatory event is planned. These include: who is being invited to join this event and what is basis of the invitation (expertise, enthusiasm, demographic characteristic or randomness); what are the terms of engagement (informing, framing, debating and deciding); and what is the scale of engagement (strategic, programmatic or personal)? They conclude that, while there is no right answer to any of these questions, having an answer is especially important for it is known that uncertainty and ambiguity on these dimensions underpins much of the dissatisfaction in practice with many participatory events and leads to serious concerns about how “genuine” they are.

## Conclusion

Rather than debating whether conceptual or methodological research is required to address the knowledge gaps, both approaches are needed and should be encouraged. Indeed the research base should be broadened even further to include the political dimension (Kieslich, Bump, Norheim, Tantivess and Littlejohns, 2016; Kieslich, Ahn, Badano, Chalkidou, Cubillos, Hauegen, Henshall, Krubiner, Littlejohns, Lu, Pearson, Rid, Whitty and Wilson, 2016). We believe that the DMAT, by investigating both process and content values, provides the pragmatic means of bringing the various stakeholders (including the “rights” and “priority” approaches) together and can facilitate the dialogue necessary to achieve fair, efficient and effective health services.

## Figures and Tables

**Table I:** The decision-making audit tool

	Description	Prompt questions	Audit question
*Process values*			
Institutional setting	Before you consider how best to respect social values and other criteria of decision making, you need first to consider the role that your organisation (or the one you are auditing) plays in the wider institutional context of health care decision making	What legal responsibilities does your organisation have with regards to health care resource allocation? What legal obligation is your organisation under to avoid discrimination, promote equality and diversity and match resources to population needs?	On a scale from 1 to 5 how sure are you that your organisation has systems in place to identify and address its legal responsibilities? (1: representing very unsure, 2: somewhat unsure, 3: undecided, 4: sure, 5: very sure)
Transparency	Those who commission health care are given considerable power and with power comes responsibility. Being transparent in their decision making is one way in which organisations can assure themselves that they are not making decisions on grounds that are considered unfair or biased by the wider public	How clearly does your organisation offer reasons for decisions? When your organisation is faced with a difficult decision, has it been open about the difficulties with those who will ultimately be affected by the decisions?	On a scale from 1 to 5 how sure are you that your organisation can demonstrate that it offers understandable and accessible reasons for its decisions? (1: representing very unsure, 2: somewhat unsure, 3: undecided, 4: sure, 5: very sure)
Accountability	Those who commission health care have a great number of people and organisations to whom they are accountable. Sometimes accountability if formal, involving legal or financial accountability. Sometimes it is less formal, for example, to colleagues or local media outlets. In all cases accountability requires an ability to give reasons for one’s decisions	Has your organisation identified to whom it is formally and informally accountable? Does your organisation provide an account of the reasons for its decisions in a variety of formats so that those who are less used to reading long and complex documents can follow them?	On a scale from 1 to 5 how sure are you that your organisation can demonstrate that it is accountable? (1: representing very unsure, 2: somewhat unsure, 3: undecided, 4: sure, 5: very sure)
Participation	Participation of stakeholders and the wider public is important because it adds to the views and values that are considered when making decisions. Enabling different groups, e.g. patients, the public, health professional and elected officials, to contribute to decision making ensures that these different views are heard and special needs are understood	Whom does your organisation include in its decision-making process and how? What is the goal of the participation method your organisation has chosen (e.g. deliberation, consultation, elicitation of public preferences)? How are the results of participation exercises incorporated in decision making and how is this communicated to the participants?	On a scale from 1 to 5 how sure are you that your organisation can demonstrate that it ensures participation of relevant stakeholders and the wider public? (1: representing very unsure, 2: somewhat unsure, 3: undecided, 4: sure, 5: very sure)
*Content values*			
Effectiveness	Effectiveness is a necessary condition for the provision of good health and social care. No one should allocate resources to forms of care that do no good or do harm. However, knowing what is effective is not easy, especially in the absence of evidence in the form of clinical effectiveness studies in some areas of health care provision	Is there a system in place to identify the evidence for the effectiveness of commissioned services? How, and by whom, is effectiveness evidence being assessed and appraised? How are decisions made in the absence of evidence (note: absence of evidence is not the same as evidence of ineffectiveness)?	On a scale from 1 to 5 how sure are you that your organisation can demonstrate that it assesses effectiveness? (1: representing very unsure, 2: somewhat unsure, 3: undecided, 4: sure, 5: very sure)
Cost effectiveness	Cost-effectiveness judgements centred around “value for money” can be controversial. For some it means that there is a risk that financial considerations could be put before patients’ needs. For others it means that the needs of all patients, rather than a few, are considered and that the best possible care for the largest number of patients is secured	Is there a system in place to identify national guidance or standards such as NICE recommendations? Have you taken steps to assure that what you are commissioning is cost effective? How are decisions made in the absence of evidence for cost effectiveness?	On a scale from 1 to 5 how sure are you that your organisation can demonstrate that it assesses cost effectiveness? (1: representing very unsure, 2: somewhat unsure, 3: undecided, 4: sure, 5: very sure)
Fairness	Fairness goes by different names. Some people talk about equity and others about human rights. In the area of health care prioritisation fairness relates to the question whether all those who use health care services are treated with equal concern and respect	How are vulnerable patient groups identified in your area and how do you ensure adequate services for these groups? Are services commissioned only on the basis of need and not on other characteristics such as age, gender, ethnicity or sexual orientation?	On a scale from 1 to 5 how sure are you that your organisation can demonstrate that it is fair to all population and patient groups on whose behalf it is commissioning services? (1: representing very unsure, 2: somewhat unsure, 3: undecided, 4: sure, 5: very sure)
Solidarity	Solidarity is the principle that “we are all in it together”. This value implies that costs for health care will be covered collectively in order to secure access to health care for individuals	Are services accessible for all, e.g. are there mechanisms in place to cover travel and other costs of access? Does your commissioning strategy create a situation in which some people have to fund elements of treatments from their own pockets in ways that are unduly burdensome?	On a scale from 1 to 5 how sure are you that your institutions can demonstrate that it addresses the social value of solidarity? (1: representing very unsure, 2: somewhat unsure, 3: undecided, 4: sure, 5: very sure)
